# Update on prognosis driven classification of pediatric AKI

**DOI:** 10.3389/fped.2022.1039024

**Published:** 2022-10-21

**Authors:** Mital Patel, Rasheed A. Gbadegesin

**Affiliations:** Department of Pediatrics, Division of Pediatric Nephrology, Duke University, Durham, NC, United State

**Keywords:** acute kidney injury, acute kidney disease, chronic kidney disease, biomarkers, prognostic model

## Abstract

Acute kidney injury (AKI) affects a large proportion of hospitalized children and increases morbidity and mortality in this population. Initially thought to be a self-limiting condition with uniformly good prognosis, we now know that AKI can persist and progress to acute kidney disease (AKD) and chronic kidney disease (CKD). AKI is presently categorized by stage of injury defined by increase in creatinine, decrease in eGFR, or decrease in urine output. These commonly used biomarkers of acute kidney injury do not change until the injury is well established and are unable to detect early stage of the disease when intervention is likely to reverse injury. The kidneys have the ability to compensate and return serum creatinine to a normal or baseline level despite nephron loss in the setting of AKI possibly masking persistent dysfunction. Though these definitions are important, classifying children by their propensity for progression to AKD and CKD and defining these risk strata by other factors besides creatinine may allow for better prognosis driven discussion, expectation setting, and care for our patients. In order to develop a classification strategy, we must first be able to recognize children who are at risk for AKD and CKD based on modifiable and non-modifiable factors as well as early biomarkers that identify their risk of persistent injury. Prevention of initial injury, prompt evaluation and treatment if injury occurs, and mitigating further injury during the recovery period may be important factors in decreasing risk of AKD and CKD after AKI. This review will cover presently used definitions of AKI, AKD, and CKD, recent findings in epidemiology and risk factors for AKI to AKD to CKD progression, novel biomarkers for early identification of AKI and AKI that may progress to CKD and future directions for improving outcome in children with AKI.

## Introduction

**Figure 1:**
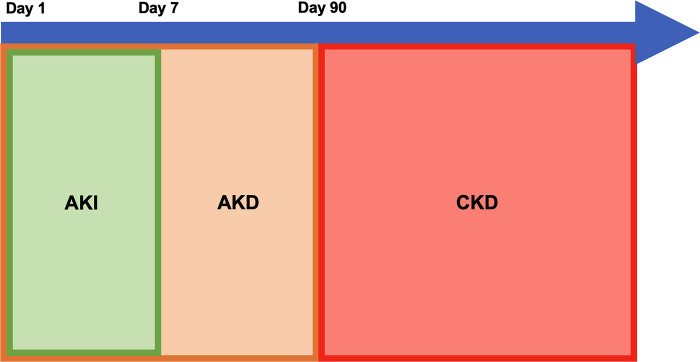
Proposed model for continuum of acute kidney injury (AKI), acute kidney disease (AKD), and chronic kidney disease (CKD).

Acute kidney injury is a very common condition affecting about 5% of non-critically ill and 27% of critically ill children (1, 2). Children who have AKI have longer hospitalizations, prolonged need for mechanical ventilation, higher risk of developing CKD especially in the setting of repeated AKIs, and increased risk of mortality (1–6). The incidence of all causes of CKD as well as CKD after AKI are not well described and is likely underreported due to lack of routine screening (7). CKD is common in the US and it is associated with poor cardiovascular outcomes, anemia, dyslipidemias, altered growth and neurocognition in children which are all associated with poor health outcomes and quality of life in both children and adults (8, 9). AKI has long been recognized as a risk factor for CKD and recent literature has suggested that acute kidney disease (AKD), a term first introduced by the KDIGO group in 2012 and defined as persistent kidney dysfunction beyond 7 days and up to 3 months, may also be an independent risk factor for CKD (10, 11). Recent literature suggests that AKI, AKD, and CKD may be a continuation of the same ongoing disease process rather than separate entities; a graphic representation of this continuum is shown in Figure 1. The trajectory of kidney recovery after AKI and AKD is highly variable and therefore it is important to better understand what factors will increase a child's risk of non-recovery and to identify at risk children early. Studies have shown that children with rapid and sustained reversal of their kidney injury within 48 to 72 h tend to have better outcomes than those with persistent kidney injury irrespective of AKI stage (12–14). Persistent, as well as relapse of reversed kidney injury, is very common in hospitalized patients and is associated with longer hospital and ICU length of stay, need for kidney replacement therapy, and mortality compared to those with early sustained reversal of AKI (6). AKD has been found to be associated with new diagnosis of CKD as well as increased 1-year mortality in adults (15). Both AKI and AKD have been shown to be independent predictors of new CKD diagnosis and by better recognizing which children are at risk for progressing along the AKI-AKD-CKD path, providers can better prognosticate and implement kidney protective measures during this vulnerable period (16–20). Although AKI as a predictor of CKD has been well studied, it is more likely that a combination of injury severity, prolonged injury, and patient level risk factors play a role in progression of AKI to AKD and CKD. Recent discoveries in risk factors for AKI to AKD progression may provide insight into which children are at risk that may benefit from closer monitoring and care by a nephrologist. By better prognosticating which children may progress along this spectrum, nephrologists would better be able to discuss methods to mitigate further kidney injury and promote kidney recovery with families and providers.

## Definitions

AKI has been well studied over the last several decades leading to significant changes in its definition and classification. The RIFLE criteria (Risk, Injury, Failure, Loss of Kidney Function, and End-stage Kidney Disease) were first proposed in 2002 the ADQI (Acute Disease Quality Initiative) group and published in 2004 (21). The RIFLE criteria were adapted for use in pediatrics (pRIFLE) and were able to accurately identify AKI in children (22). These criteria staged kidney injury severity based on serum creatinine, eGFR changes, and urine output and classified patients on two outcomes (loss of function and end-stage kidney disease). Although this criteria was able to identify patients with AKI well and to accurately prognosticate mortality it had limited evaluation in prospective studies (21). In 2005, the AKIN (Acute Kidney Injury Network) Working Group proposed a modification of the RIFLE criteria. The AKIN classification only considers a diagnosis of AKI once dehydration is corrected and urinary obstruction is ruled out. It relies on serum creatinine and urine output measurements over a 48-hour time frame and unlike the RIFLE criteria does not consider the eGFR. Much like the RIFLE criteria, the AKIN criteria was able to accurately identify patients with AKI but it had poor ability to predict in-hospital mortality (21). Most recently, the KDIGO (Kidney Disease Improving Global Outcomes) group proposed in 2007 and published in 2012 a new consensus classification system for AKI with the goal of harmonizing pediatric and adult AKI classifications (23). These definitions are now routinely used in both clinical practice and research. Table 1 shows the different criteria used by the RIFLE, pRIFLE, AKIN, and KDIGO groups for diagnosis of AKI.

**Table 1:** AKI criteria by the RIFLE, pRIFLE, AKIN, and KDIGO groups.

RIFLE			pRIFLE			AKIN			KDIGO		
Criteria	Creatinine definition	Urine output	Criteria	Creatinine definition	Urine output	Criteria	Creatinine definition	Urine output	Criteria	Creatinine definition	Urine output
Risk	>/= 1.5-fold increase from baseline SCr or decrease in eGFR >/= 25%	<0.5 ml/kg/hr × 6 h	Risk	Decrease in eGFR by >/= 25%	<0.5 ml/kg/hr × 8 h	Stage 1	>/= 1.5-fold or >/= 0.3 mg/dl increase from baseline SCr	<0.5 ml/kg/hr × 6 h	Stage 1	1.5–1.9-fold increase or >/= 0.3 mg/dl increase from baseline SCr	<0.5 ml/kg/hr × 6 h
Injury	>/= 2-fold increase from baseline SCr or decrease in eGFR >/= 50%	<0.5 ml/kg/hr × 12 h	Injury	Decrease in eGFR by >/= 50%	<0.5 ml/kg/hr × 16 h	Stage 2	>/= 2-fold increase from baseline SCr	<0.5 ml/kg/hr × 12 h	Stage 2	2-2.9- fold increase from baseline SCr	<0.5 ml/kg/hr × 12 h
Failure	>/= 3-fold increase from baseline SCr or decrease in eGFR >/= 75%	<0.3 ml/kg/hr × 24 h or anuria × 12 h	Failure	Decrease in eGFR by >/= 75%	<0.3 ml/kg/hr × 24 h or anuria × 12 h	Stage 3	>/= 3-fold increase from baseline SCr or SCr >/=4 mg/dl with an acute rise of >0.5 mg/dl or initiation of RRT	<0.3 ml/kg/hr × 24 h or anuria × 12 h	Stage 3	>/= 3-fold increase from baseline SCr or SCr >/= 4 mg/dl with an acute risk of >0.5 mg/dl or initiation of RRT	<0.3 ml/kg/hr × 24 h or anuria × 12 h
Loss	Persistent loss of kidney function for >4 weeks		Loss	Persistent loss of kidney function for >4 weeks							
ESKD	End stage kidney disease		ESKD	End stage kidney disease							

AKD is a newer concept in the kidney disease lexicon and was proposed to better define the time period between AKI and CKD. The first concept was first suggested in 2012 and the first suggested definition and staging guidelines for AKD were recommended by the ADQI group in 2017. The ADQI group defined AKD as persistent stage 1 AKI or greater for >/= 7 days and staged it based on degree of serum creatinine increase from baseline (24). This definition was broadened in 2021 by the KDIGO group to include functional criteria for AKI as well as more subtle changes in eGFR and creatinine that may not meet the criteria for diagnosis of AKI (11). AKD can further be classified as AKD with or without AKI which includes those patients who don't meet creatinine or urine output criteria for AKI but do meet criteria for AKD (11, 24). Although no specific staging was presented in the consensus guidelines, it was proposed that AKD could be staged based on severity of eGFR decline as well as albuminuria (11). The ADQI and KDIGO definitions of AKD are outlined in Table 2.

**Table 2:** ADQI and KDIGO definitions for diagnosis of AKD.

ADQI		KDIGO	
Criteria	Definition	Criteria	
Subacute (stage 0)	C: SCr not back to baseline B: Biomarker or loss of kidney reserve A: No evidence of injury	Functional criteria	Meeting AKI definition OR GFR <60 ml/min/1.73 m^2^ OR decrease in GFR by >/= 35% OR increase in SCr by >50%
Stage 1	SCr 1.5-fold increase from baseline	Structural criteria	Marker of kidney damage (albuminuria, hematuria, or pyuria)
Stage 2	SCr 2-fold increase from baseline	Proposed G and A staging	Increasing severity based on decreasing eGFR and/or degree of albuminuria
Stage 3	SCr 3-fold increase from baseline or need for RRT		

Lastly, CKD is defined as an abnormality of kidney function defined by eGFR <60 ml/min/1.73 m^2^ or abnormal structure or function present for >90 days with implications on future health. Markers of kidney damage can include albuminuria, abnormal urine sediments, electrolyte or other abnormalities suggesting tubular disorder, abnormal kidney histology, abnormal kidney imaging, or history of kidney transplant. CKD, like AKI, is classified into stages by severity of the kidney dysfunction by eGFR and degree of albuminuria originally outlined by the Kidney Disease Outcomes Quality Initiative (KDOQI) group and most recently defined by the KDIGO group [Table 3] (25, 26).

**Table 3:** KDIGO criteria of CKD stages.

KDIGO			
G Criteria	eGFR definition	A Criteria	Albuminuria range
G1	>/= 90	A1	<30 mg/g or <3 mg/mmol
G2	60–89	A2	30–299 mg/g or 3–29 mg/mmol
G3a	45–59	A3	>/= 300 mg/g or >/= 30 mg/mmol
G3b	30–44		
G4	15–29		
G5	<15		

The focus of the remainder of this review will be on the current data on the prevalence of AKD, and the risk factors for AKI-AKD and AKD-CKD progression. The review will also describe the state of knowledge on candidate biomarkers for disease progression following AKI and how we can leverage these biomarkers and clinical factors to identify children that are at risk of disease progression.

## Epidemiology of AKD

AKD is a relatively new concept and there are limited studies on the prevalence of AKD in pediatric populations. Table 4 outlines pediatric and adult studies of AKD epidemiology, risk factors, and outcomes. Pediatric studies in special populations have shown variable incidence of AKD ranging from 6.2% in children undergoing cardiopulmonary bypass, 13% in non-kidney solid organ transplant recipients, 15.6% in children with severe malaria infection, 35.3% in allogeneic hematopoietic stem cell recipients, and 42.3% in hospitalized children with AKI (16, 27–30). Adult studies have shown even more variability with incidence ranging from 3.8% in the general adult population of a single Canadian territory to 4.4%–74% in adults of special populations including those undergoing cardiac and non-cardiac procedure, chemotherapy, hematopoietic stem cell transplant recipients, those with AKI, heart failure, sepsis, cirrhosis, and SARS-CoV-2 infection (20). Hospitalized adults with AKI admitted to both the general floors as well as ICUs had high prevalence of AKD with most studies showing about one-fourth to half of all patients developing AKD (19, 31–40). Adults with cardiac diseases, post cardiac surgery, sepsis and cirrhosis all had high AKD prevalence suggesting hemodynamic status may play a significant role in sustained kidney injury and progression (17, 18, 37–46, 48). The wide variability in prevalence suggests there is a significant role for patient level and hospital acquired risk factors in the development of AKD after AKI.

**Table 4:** Prevalence, risk factors, and outcomes of AKD in pediatric and adult studies.

Author	Age Group	Patient Population	N	AKD prevalence	AKI-to-AKD risk factors	Outcomes of AKD
Namazzi et al. 2022 (27)	Pediatric	severe malaria	598	15.6%	AKI stage, blackwater fever, high risk NGAL level	Increased mortality post discharge
Daraskevicius et al. 2020 (28)	Pediatric	allogeneic hematopoietic stem cell transplant recipients	51	35.3%	Age, higher baseline eGFR, BMI	
Patel et al. 2021 (16)	Pediatric	non-kidney solid organ transplant recipients	338	13%	AKI stage	New onset CKD
Deng et al. 2019 (29)	Pediatrics	AKI, hospitalized	990	42.3%	AKI stage, glomerulonephritis	Increased risk of mortality, persistent kidney dysfunction, or new RRT at 30 and 90 days
LoBasso et al. 2022 (30)	Pediatrics	AKI, Cardiopulmonary bypass	701	6.3%		Increased mortality
James et al. 2019 (20)	Adult	General population, Alberta, CA	1,109,099	3.8%		Increased risk of new CKD, CKD progression and mortality
Xiao et al. 2020 (31)	Adult	Hospitalized, AKI	2,556	53.17%	AKI stage, Gender, hepatorenal syndrome, intrinsic kidney disease, oligo/anuria, respiratory failure, elevated BUN	Increased 90-day mortality
Zhao et al. 2021 (32)	Adult	Hospitalized, AKI	261	68.6%	Pre-existing diabetes, anemia, oliguria, peak creatinine	
Hsu et al. 2022 (33)	Adult	Hospitalized, AKI	310	77.1%	AKI stage, Nephrotoxin exposure within 1 week of AKI, early dialysis requirement	Increased 30-day mortality and lower dialysis-independent survival
Nagata et al. 2018 (34)	Adult	Hospitalized, AKI	7,582	13.8%		
Lertussavavivat et al. 2022 (19)	Adult	Hospitalized	9,800	7%–8%		Increased in hospital mortality and new CKD
Andonovic et al. 2022 (35)	>16 years old	ICU	1,620	24.9%	Male sex, sepsis, lower baseline eGFR	Increased ICU mortality and MAKE
Rimes-Stigare et al. 2018 (36)	Adult	AKI, ICU	336	18.9%		
Gameiro et al. 2021 (37)	Adult	AKI, sepsis, ICU	252	53.9%		Increased need for chronic dialysis, eGFR decline, and mortality after hospital discharge
Flannery et al. 2021 (38)	Adult	ICU, sepsis	6,290	46.9%		Increased risk of CKD and ESKD
Peerapornratana et al. 2020 (39)	Adult	ICU, sepsis	598	26.9%	Male sex, African American race, pre-existing CKD	Increased hospital length of stay
He et al. 2021 (40)	Adult	ICU, sepsis	209	5.5%		
Chen et al. 2022 (41)	Adult	Acute decompensated heart failure	7,519	21.2%	AKI stage, female sex, pre-existing diabetes, CKD, hemoglobin, albumin, BNP, inotrope use during admission, outpatient diuretic use	Increased risk of mortality, MAKE, and hospitalization for heart failure
Hsu et al. 2020 (42)	Adult	ECMO	168	55.4%		Increased risk of mortality on ECMO
Mizuguchi et al. 2018 (43)	Adult	Post cardiac surgery	10,234	4.4%–4.8%	AKI stage, age, gender, and surgery type	Increased risk of kidney replacement therapy and mortality
Matsuura et al. 2020 (44)	Adult	Post cardiac surgery	3,605	38.6% in those with transient AKI (<3 days); 74.1% in those with AKI		Increased risk of 90-day mortality and persistent kidney function decline
Chen et al. 2020 (45)	Adult	Coronary care unit	269	47.6%	Age, hemoglobin, ejection fraction, serum IL-18	
Kofman et al. 2019 (17)	Adult	Myocardial infarction, primary percutaneous intervention	225	36%	Higher peak serum creatinine, age	Increased risk of mortality
Shaw et al. 2022 (46)	Adult	Post noncardiac surgery	112,912	2.8%	Intraoperative hypotension	Increased hospital length of stay, readmission ate, and 7-day new onset RRT need
Chen et al. 2022 (47)	Adult	Post cardiac surgery	5,127	7.2%	Intraoperative venous congestion	
Liu et al. 2022 (48)	Adult	Post coronary angiography	9,223	16.7%		Increased risk of mortality
Tonon et al. 2021 (18)	Adult	Cirrhosis	272	29.4%	Age, lower eGFR at study entry, lower serum albumin, sodium, and hemoglobin	Increased risk of hospitalization, complications of cirrhosis, and mortality
Fernandes et al. 2022 (49)	Adult	Cisplatin, carboplatin or oxaliplatin exposure	212	7%		Increased risk of mortality
Rachman et al. 2021 (50)	Adult	Carboplatin, nasopharyngeal carcinoma	120	38.4%		
Mima et al. 2019 (51)	>16 years old	Hematopoietic stem cell transplant recipients	108	15.7%	ABO incompatible transplant, higher incidence of graft versus host disease	
Wu et al. 2018 (52)	Adult	Total knee arthroplasty	458	3.3%	Age, history of coronary artery disease	
Marques et al. 2021 (53)	Adult	SARS-CoV-2 infection	544	25.7%	Pre-existing hypertension, CKD, lower hemoglobin, lower CRP, nephrotoxin exposure	Increased risk of in- hospital mortality
Sindhu et al. 2022 (54)	Adult	SARS-CoV-2 infection, AKI, no baseline CKD	115	40.9%	Pre-existing hypertension, severe albuminuria	
Sarwal et al. 2022 (55)	Adult	SARS-CoV-2 infection, AKI	1,074	28.2%	Baseline CKD >/= stage 3, congestive heart failure, ongoing diarrhea	

Risk factors for AKD in children have not been widely studied. Few studies that have been published demonstrated a higher risk of AKD in children with higher stages of AKI with one study showing that stage of AKI predicted AKD in a graded manner (16, 27, 29). Though stage of AKI has been shown to be an important risk factor for progression to AKD, studies in adults and children have shown patient-level and acquired risk factors independently increased risk of AKD independent of AKI stage suggesting that patients with lower stages of AKI also deserve close evaluation for AKD risk. A study of children who have undergone hematopoietic stem cell transplant showed that older age, higher baseline eGFR and higher body mass index (BMI) were associated with increased risk of AKD (28). A study of a more general population of hospitalized children also showed that children with glomerulonephritis compared to other AKI etiologies were at higher risk for developing AKD after AKI suggesting that intrinsic kidney disease may also be an important predictor of progression to AKD (29). Although this is the only study assessing AKI etiology as a risk factor for AKD in pediatrics, a study in adults hospitalized with AKI showed similar results when evaluating intrinsic kidney injury and rates of AKD suggesting that AKI etiology is also an important consideration when evaluating a patient's prognosis for kidney recovery (31).

There have been more comprehensive studies of risk factors for AKD development in adults as outlined in Table 4. In hospitalized adults with AKI, age, gender, intrinsic kidney disease, AKI stage, oliguria, anuria, nephrotoxin exposure, early need for dialysis, BUN level, diabetes, anemia, and respiratory failure have been associated with AKD development (31–33). In adults admitted to an ICU setting age, male sex, African American race, sepsis, lower baseline eGFR and CKD, anemia, and lower ejection fraction have been shown to be risk factors for AKD (35, 39, 45). In the setting of cardiac disease including myocardial infarction, heart failure, and cardiac surgeries, increased risk of AKD was associated with age, female sex, AKI stage, diabetes, baseline CKD, hemoglobin, albumin, BNP, inotrope use, and outpatient loop diuretic use (17, 41, 43, 47). There are several established risk factors for developing AKD in adults undergoing non-cardiac procedures including age, intraoperative hypotension, and coronary artery disease (46, 52). In addition, other risk factors in adults hospitalized with COVID include hypertension, baseline CKD ≥ stage 3, low hemoglobin, low CRP, nephrotoxin exposure, and congestive heart failure have been associated with AKD (53–55). These studies demonstrate that there are some risk factors for AKD that are common to different populations but in general, each special population has its own unique patient-level risks. This highlights the need for comprehensive and large cohort studies to define the risk factors associated with AKD and develop a better understanding of how these risk factors play a role in prolonged kidney injury. No comprehensive studies have been completed yet that have assessed a wide range of patient-level risk factors for AKD in children with AKI.

Outcome studies in both adults and children have shown that AKD and not AKI stage is a significant risk factor for new onset CKD, need for kidney replacement therapy, and mortality further underscoring the importance of understanding patient level risk factors associated with development of CKD following AKI. The variability in AKD epidemiology suggests a significant relationship between AKI stage, patient level pre-existing risk factors, modifiable risk factors including medication exposures and hemodynamic status, as well as etiology of AKI. A risk prediction tool utilizing these different exposure patient-level and iatrogenic criteria may better allow for clinical risk stratification and identification of patients who are likely to develop AKD. This significant variability in prevalence of AKD as well as risk factors is shown in Table 4 and further emphasizes the fact that different clinical tools may be needed for separate populations.

### CKD after AKD

The prevalence of CKD is significantly higher in adults with AKD than those without and prevalence has also been shown to increase with increasing AKD stage as defined by the ADQI group (17–19, 33, 38). One study demonstrated that AKD was associated with higher risk of new CKD, progression of pre-existing CKD, and ESKD (20). However, prevalence of CKD after AKD is not well studied in children with only one study showing the odds of CKD after AKD was 29-fold higher (16). More studies are needed in children to understand the risk of CKD after AKD and to assess which patient level risk factors increase the risk of progression from AKD to CKD.

## Biomarkers for AKI

Common biomarkers of AKI including creatinine, cystatin C, and urine output lack the ability for early kidney injury detection. Many other biomarkers have been studied with the goal of early identification of AKI but there have been minimal studies assessing their value as predictive markers for progression of AKD to CKD. A summary of well-studied candidate biomarkers is reviewed here and reviewed in Table 5.

**Table 5:** Candidate biomarkers for risk stratification along the AKI-AKD-CKD path.

Candidate Biomarker	Source	Initial increase	Peak level	Proposed cut off value for identifying increased risk of persistent AKI or AKD	Increased in AKD	Increased in CKD	Possible predictor of AKI, AKD, CKD progression?
NGAL	Thick ascending loop of Henle and collecting duct	1–3 h after injury	4–6 h after injury	>/= 300 ng/ml for unresolved AKI	Yes	Yes	Yes
KIM-1	Proximal tubule	Within hours of injury	18–24 h after injury	Variable based on AKI etiology	Unknown	Yes	Yes
IL-18	Tubular epithelial cells	4–6 h after injury	12 h after injury	400 ng/ml for increased risk of AKD	Yes	Yes	Yes
[TIMP-2] × [IGFBP7]	TIMP-2: distal nephron; IGFBP7: proximal tubule	Within 4 h of injury	4 h after injury	>0.3 ng/ml	Yes	Yes	Yes
TNFR-1 and TNFR-2	TNFR-1: Most cells; TNFR-2: endothelial cells, fibroblasts, neurons, myeloid cells, and T- and B-cell subsets	Unknown	Unknown	Unknown	Unknown	Yes	Yes
L-FABP	Proximal tubule epithelial cell cytoplasm	Within 2 h of injury	3 h after injury	>17.4 micrograms/gram creatinine as a marker for increased risk of kidney function decline	Unknown	Yes	Yes

### NGAL

Neutrophil gelatinase associated lipoprotein (NGAL) is a 25 kDa protein that is part of the lipocalin family. NGAL prevents iron uptake by bacteria allowing it to play a role in the innate immune system (56). Within the kidneys, NGAL is thought to be produced by the thick ascending loop of Henle and the intercalated cells of the collecting duct. Kidney damage is thought to lead to decreased reabsorption by the proximal tubule which may lead to increased urinary NGAL (57). Ischemic and toxic AKI highly upregulate NGAL expression and there is an increase in detectable levels as early as 2–3 h after injury and up to 5 days after initial injury (58, 59). The ability to use NGAL for early diagnosis of AKI was first studied by Mishra et al. in 2005 who noted that in 71 children undergoing cardiopulmonary bypass, a well-known risk factor for AKI, urine, and serum NGAL quickly rose within 2 h after the bypass procedure in children who later developed AKI. The rise in NGAL levels was rapid compared to the delayed rise in serum creatinine that occurred about 1–3 days after the procedure. NGAL was also found to be elevated, but to a lesser extent, in those without creatinine based AKI diagnosis, identifying possible subclinical injury (59). This highlighted the value of NGAL in the early diagnosis of AKI. Since then NGAL has been studied in children and adults that are critically ill, undergoing cardiac surgery, transplant, and presenting to the emergency department (60). Though NGAL has been shown to be a relatively good early predictor of kidney disease in these populations, there is significant variability in the AUC-ROC making it difficult to establish set cut offs for the diagnosis of kidney injury. There have been limited studies on the ability of NGAL to predict AKD with one study showing that higher concentration of NGAL was independently associated with longer duration of AKI (≥ 7 days) (61) and a second study showing that children with a NGAL level ≥300 ng/ml at the time of admission were more likely to develop AKD (27). Serum NGAL levels have been shown to be elevated in children and adults with CKD and that it may be a predictor of CKD progression (62, 63) possibly making it a good biomarker of persistent kidney damage. It is speculated that NGAL levels increase in proportion to tubular damage, neutrophil activation or inflamed vasculature which may explain elevated levels in CKD. Decreased clearance in the setting of CKD is hypothesized to prevent proximal tubule reabsorption of NGAL which may lead to increased serum levels (62). More studies are needed to assess the ability of early NGAL elevation to predict risk of AKD and CKD.

### KIM-1

Kidney Injury Molecule-1 (KIM-1) is a 39 kDa transmembrane protein with an immunoglobulin domain and mucin domain that is expressed in the proximal tubule (64, 65). Expression of KIM-1 is upregulated in the setting of ischemia-reperfusion injury and the ectodomain of KIM-1 is shed from cells and can be detected in the urine and blood of patients with AKI within hours of kidney injury (66–71). KIM-1 has also been shown to be a predictor of AKI in the setting of nephrotoxin exposure (72, 73). Elevated urine KIM-1 levels can persist after a patient has had apparent recovery from AKI suggesting it is a marker of subclinical and ongoing injury (74). It is thought that KIM-1 has better diagnostic accuracy in pediatric patients than adults due to higher level of comorbidities in the latter as well as normal increase in KIM-1 levels with age (67, 75). Similar to NGAL, there are not well established set cut offs for KIM-1 for the diagnosis of kidney damage (68). KIM-1 is also a sensitive biomarker for CKD because it is thought to play a role in proinflammatory state and kidney fibrosis (76). The usefulness of KIM-1 in the diagnosis and prediction of AKD is unknown and further studies are warranted. A summary of KIM-1 and its potential role as an early biomarker of long-term injury is presented in Table 5.

### IL-18

Interleukin-18 (IL-18) is a proinflammatory cytokine (77). Within the kidney it is expressed in the tubular epithelial cells and is stored intracellularly as its inactive form, pro-IL-18 (78). Once cleaved by caspase-1, it is secreted as its biologically active form IL-18 which acts on the IL-18 receptor to cause downstream upregulation of pro-inflammatory gene transcription (78). IL-18 levels have been shown to be elevated prior to AKI development (79, 80) and are predictors of AKI progression in the setting of critical illness (81–84). Levels increase about 6 h after injury and peak at about 12 h (71). In children, IL-18 has been demonstrated to be a predictor of AKI in preterm infants but there are no well-established cutoffs for diagnosis of AKI (85–88). The AUC-ROC varies significantly and IL-18 has low sensitivity but high specificity indicating that some patients with AKI may not have elevated IL-18 levels (89). IL-18 can remain elevated for 41 months to 7 years in children with AKI post cardiac surgery and a study of adult patients showed that significantly elevated IL-18 levels were associated with longer term eGFR decline suggesting that it may be a marker of ongoing damage (90–92). Lastly, one study of adults admitted to a cardiac care unit showed that IL-18 was independently associated with development of AKD (45). In this study by Chen et al., patients with AKD had mean serum IL-18 levels of 454 ng/ml (SD 36 ng/ml) compared to 256 ng/ml (SD 15 ng/ml) in the non-AKD group and based on this, a possible cutoff value of 400 ng/ml was suggested as a marker for increased risk of AKD, this finding warrants further studies (45). IL-18 is also upregulated in CKD and may play a role in tubulointerstitial fibrosis by inducing tubuloepithelial cell injury and activation (93). These findings are summarized in Table 5. Given this finding of more elevated levels in those with AKD as well as its role as a marker of ongoing inflammation, IL-18 may be a good early predictor of who is at risk of persistent kidney disease.

### [TIMP-2]× [IGFBP7]

Tissue inhibitor of metalloproteinases 2 (TIMP2) and insulin-like growth factor-binding protein 7 (IGFBP7) are involved in cell-cycle arrest preventing cells from dividing until damage can be repaired and therefore are markers of cell stress (94). They are upregulated in ischemia, sepsis, oxidative stress, and exposure to toxins (95). These biomarkers can be found in the urine within hours of kidney injury (96–98). The combination of these two biomarkers was shown to have improved risk stratification and predicts AKI progression in critically ill patients (81, 96, 99–101). When paired with a clinical model they have improved risk prediction for AKI (96). These biomarkers have been shown to be good predictors of AKI even in the setting of common comorbid conditions including CKD, congestive heart failure, and diabetes mellitus (102). In critically ill patients there was a seven fold increase in risk of AKI in patients with a [TIMP-2]× [IGFBP7] value of >0.3 ng/ml when compared to those with a score ≤0.3 ng/ml (99) making this a possible acceptable cut off for assessing for increased AKI risk in critically ill patients. The utility of this biomarker and relative cut offs for AKI risk prediction are variable in non-critically ill populations (103). Conflicting studies have shown variable utility of [TIMP-2]× [IGFBP7] in predicting persistent kidney injury beyond 3–5 days. A study of adults admitted to a single ICU demonstrated that the ([TIMP-2]× [IGFPB7]/1000) measured at hours 0, 4, 12, and 24 of ICU admission had poor ability to predict persistent (non-recovery at 5 days) compared to transient AKI (104). Another study of adults admitted to 11 different ICUs with sepsis demonstrated that patients with higher ([TIMP-2]× [IGFPB7]/1000) values early in their course were more likely to develop persistent AKI (non-recovery at 3 days) but that the short term changes in the biomarkers over the first 24 h failed to similarly identify at risk patients (105). Lastly, a retrospective cohort study of adults also admitted to an ICU who had ([TIMP-2]× [IGFPB7]/1000) and procalcitonin measured at the time of admission demonstrated that those with a positive double biomarker test had a higher risk of developing AKD (106). These studies done by Titeca-Beauport et al. and Godi et al. suggest that early, rather than sustained elevation or trajectory, of [TIMP-2]× [IGFBP7] may be a better early marker of AKD risk. Lastly, this biomarker in mouse models has also been shown to predict predisposition for repeat AKI episodes, and in a study of adults, elevated [TIMP-2]× [IGFBP7] at the time of AKI was predictive of CKD development even after complete recovery from AKI (107, 108). The possible utility of [TIMP-2]× [IGFBP7] for early risk stratification of at-risk patients is summarized in Table 5.

### TNFR-1 and TNFR-2

Tumor necrosis factor receptor-1 (TNFR-1) and tumor necrosis factor receptor-2 (TNFR-2) are soluble markers of neutrophil-endothelial cell interaction (109) and play a role in apoptosis (110). TNFR-1 is expressed in most cells and is activated by membrane bound and soluble TNFα whereas TNFR-2 is present only in endothelial cells, fibroblasts, neurons, myeloid cells, and T- and B-cell subsets and is only activated by membrane bound TNFα. As a result of cellular activation by TNFα, alternative splicing of mRNA occurs resulting in the production of soluble TNFR which is detectable in the blood (110). Serum TNFR-1 and -2 levels have been shown to be independent predictors of AKI, severe AKI, need for kidney replacement therapy, and mortality and elevated TNFR-1 has been shown to be a marker of slower kidney recovery (109, 111–115). In a study of patients with COVID-19, patients were found to have increasing levels of TNFR-1 and TNFR-2 with increasing stage of AKI (114). Lastly, TNFR-1 and-2 have been shown to be elevated in adults with diabetes associated proteinuria, hypertension, CKD and ESKD and more recently in non-diabetic adults as well (116–119). TNFR levels are associated with degree of interstitial fibrosis in IgA nephropathy likely related to TNFα release from mesangial cells and subsequent inflammation (120). This may indicate that elevated levels of soluble TNFR-1 and -2 in the setting of CKD may be indicators of ongoing inflammation within the kidney. No studies have shown a clear timing of detectable levels, peak levels, or cutoff value for identifying AKI or increased risk of AKD. Known data regarding these two biomarkers are summarized in Table 5. TNFR-1 and TNFR-2 have not been studied for its ability to predict AKD, but it may be a good candidate biomarker to monitor overtime to assess for ongoing inflammation and AKD risk.

### L-FABP

Liver fatty acid binding protein (L-FABP) is found in the cytoplasm of proximal tubule epithelial cells and is involved in free fatty acid transportation (121). This segment of the nephron is highly dependent on fatty acid metabolism for energy production and normal cellular transport functions. L-FABP levels have been shown to be increased in the setting of kidney hypoxia and ischemia (121, 122). Mouse models have shown detectable increase in L-FABP levels within 2 h of ischemia-reperfusion or nephrotoxic medication exposure with peak levels at 3 h. From there L-FABP levels steadily decreased but remained detectable 24 h after reperfusion (123). L-FABP levels have been found to be elevated in adults with various etiologies of AKI as well as in children undergoing cardiac surgery in whom levels increase as early as 4 h after their procedure (124–126). Elevated levels have also been associated with increased risk of AKI non-survival and development of CKD (125, 127). Similar to TNFR-1 and -2, there are no established cutoff values though a urine value of greater than 17.4 micrograms/gram creatinine has been associated with more significant deterioration of kidney function in patients with CKD (127). Though it has not been studied for its ability to predict risk of AKD, it may represent a good biomarker as it may indicate ongoing abnormalities in metabolism and energy production; further studies to delineate its role in predicting AKD and CKD risk are needed.

## Perspectives and future directions

Severe AKI and repeated AKIs have long been known to be a risk factor for CKD but recent literature suggests that AKD may be better predictor for disease progression. Limited studies in children and many studies in adults have shown that risk of AKD is dependent on stage and duration of AKI as well as a myriad of patient level risk factors. AKD itself increases the risk of new onset CKD, progression of CKD, development of ESKD, and mortality. By better understanding which pre-existing and iatrogenic factors place children with AKI at risk for longer duration of kidney injury we can help better prognosticate their likelihood of AKD and CKD after AKI. Early identification of at-risk children can allow for better kidney centered care and avoidance of repeated injuries and factors that promote irreversible kidney injury.

**Figure 2:**
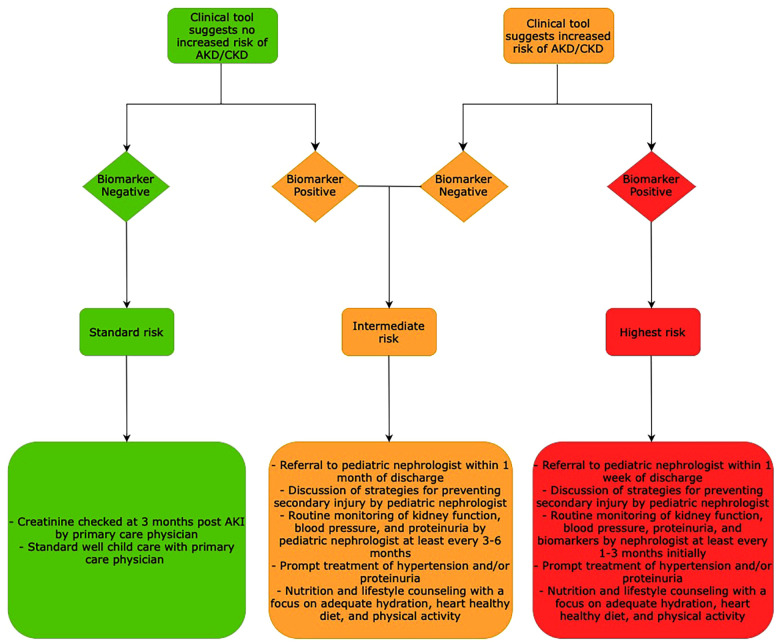
Proposed schematic for biomarker and clinical tool-based prognostication for AKI-AKD-CKD progression and follow up nephrology care post AKI.

We propose that a combination of a clinical tool which identifies AKD and CKD risk by clinical factors in conjunction with biomarkers of early and prolonged kidney injury would best allow nephrologists to identify children who are at risk for persistent kidney injury. A proposed schematic for biomarker and clinical tool-based care is shown in Figure 2 with suggested follow up, counseling, and monitoring adapted and extrapolated from the recent ADQI meeting report (128). This model has been extrapolated to include monitoring for development of AKD as there are no specific guidelines or models in the literature for this. As shown in this review, there is significant heterogeneity in the risk of kidney disease progression as well as risk factors among both children and adults. In this setting, different clinical tools tailored to individual populations may be more suitable rather than one universal clinical risk prediction tool. We have suggested candidate biomarkers in this article which should be better studied for their ability to predict risk of progression to AKD and CKD. These biomarkers tend to be altered much sooner than classic markers of AKI including serum creatinine, urine output, and cystatin C. Many of these biomarkers have been shown to be persistently elevated long after AKI has resolved underscoring the importance of identifying molecular markers that can reveal subclinical and prolonged kidney dysfunction. There are limited studies that have shown that elevation of these biomarkers in the AKI period may aid in identification of children who are at risk for AKD. Further studies are needed to clarify if early changes in biomarkers can be used in prognostication.

Although it is important to classify AKI by stage, it is also important to recognize that this alone does not explain a child's risk of developing CKD and its sequelae. Other factors must be taken into consideration when assessing the overall morbidity and mortality associated with a single or even repeated episodes of AKI. It may be more appropriate to consider how the child's future kidney health will be affected by their AKI episode and for this we must better understand the other risk factors that promote AKI-AKD-CKD progression. There are no well-studied therapeutic options for preventing kidney disease progression after AKI but by better understanding which children are at risk we may facilitate the study of disease modifying behaviors and therapies. Despite all that is known, more studies are needed for early identification of at-risk children so we may better mitigate adverse outcomes of AKI in this vulnerable population.
